# Quantifying harms to others due to alcohol consumption in Germany: a register-based study

**DOI:** 10.1186/s12916-019-1290-0

**Published:** 2019-03-19

**Authors:** Ludwig Kraus, Nicki-Nils Seitz, Kevin D. Shield, Gerrit Gmel, Jürgen Rehm

**Affiliations:** 1 IFT Institut für Therapieforschung, Leopoldstraße 175, 80804 Munich, Germany; 2 Department for Public Health Sciences, Stockholm University, Sveavägen 160, Sveaplan, 10691 Stockholm, Sweden; 3 Institute of Psychology, ELTE Eötvös Loránd University, Kazinczy utca 23-27, 1075 Budapest, Hungary; 4 Centre for Addiction and Mental Health, Institute for Mental Health Policy Research, 33 Russell Street, Toronto, ON M5S 2S1 Canada; 5 Centre for Addiction and Mental Health, Campbell Family Mental Health Research Institute, 33 Russell Street, Toronto, ON M5S 2S1 Canada; 6 Dalla Lana School of Public Health, University of Toronto, 155 College Street, 6th floor, Toronto, ON M5T 3M7 Canada; 7 Faculty of Medicine, Institute of Medical Science, University of Toronto, Medical Sciences Building, 1 King’s College Circle, Room 2374, Toronto, ON M5S 1A8 Canada; 8 Department of Psychiatry, University of Toronto, 250 College Street, 8th floor, Toronto, ON M5T 1R8 Canada; 9 Institute of Clinical Psychology and Psychotherapy and Center of Clinical Epidemiology and Longitudinal Studies (CELOS), Technische Universität Dresden, Chemnitzer Str. 46, 01187 Dresden, Germany

**Keywords:** Alcohol, Harm to others, Fetal alcohol syndrome (FAS), Fetal alcohol spectrum disorders (FASD), Road traffic fatalities, Interpersonal violence

## Abstract

**Background:**

The consumption of alcohol increases the risk of drinkers harming others. The extent of alcohol’s morbidity and mortality harms to others in Germany in 2014 was estimated for (1) fetal alcohol syndrome (FAS) or fetal alcohol spectrum disorders (FASD) among newborns, (2) road traffic fatalities, and (3) interpersonal violence-related deaths.

**Methods:**

The incidences of FAS and FASD were estimated by means of a meta-analytical approach, combining data on alcohol use during pregnancy and the risk relationship between alcohol consumption during pregnancy and FAS/FASD. In order to estimate alcohol-attributable road traffic fatalities and interpersonal violence due to the drinking of others, an attributable fraction methodology was applied to cause-of-death statistics for road traffic and interpersonal violence-related deaths.

**Results:**

For 2014, the incidences of FAS and FASD were estimated at 41 children per 10,000 live births (95% CI 24; 63) and 177 children per 10,000 live births (95% CI 135; 320), or 2930 (95% CI 1720; 4500) and 12,650 (95% CI 9650; 23,310) children, respectively. Furthermore, alcohol was estimated to be responsible for 1214 (95% CI 1141; 1287) third-party road traffic fatalities and 55 (95% CI 46; 64) deaths from interpersonal violence, representing 45.1% of all third-party road traffic fatalities and 14.9% of all interpersonal violence deaths.

**Conclusion:**

These study’s estimates indicate there is a substantial degree of health harm to third parties caused by alcohol in Germany. While more research on harms to others caused by alcohol is needed to provide comprehensive estimates, the results indicate a need for effective prevention.

## Background

Alcohol is a leading risk factor for the burden of disease and injury as well as for premature mortality globally, and in particular in Western Europe and Germany [1]. However, current alcohol-attributable harm estimates include mainly health harms to the individuals who consume alcohol despite the harm from alcohol consumption extending to people other than the drinker. For instance, traffic collisions caused by drinking drivers may involve and injure passengers, other drivers, or pedestrians, irrespective of whether or not these individuals had consumed alcohol. Sober people walking home at night may be harassed and injured by drunken pedestrians or may be attacked while attempting to separate intoxicated young people who are fighting. Children may suffer problems caused by a drinking father or mother, and continued heavy drinking during pregnancy may severely harm the health of the newborn. In a seminal contribution to this field of research, Nutt and colleagues [2] concluded that if both harm to the user and harm to others were accounted for, alcohol was the most harmful psychoactive substance in the UK in 2010, followed by heroin and crack cocaine. Furthermore, of all the substances compared, alcohol was the greatest contributor to harms to others.

In general, health records based on the International Classification of Diseases (ICD) focus on the characteristics of the individual affected by the disease conditions. Particularly in the context of injuries, which in many instances involve an interaction with others, the diagnostic codes pay attention to the circumstances, for instance, whether the injury occurred as the result of a car crash or fire; however, the diagnostic codes usually neglect the involvement of other people and their characteristics. Additionally, in surveys regarding alcohol-related problems, only the interviewee’s own drinking and its consequences are commonly recorded, although positive answers to questions concerning whether the respondent’s drinking has caused family, marital, or workplace problems clearly indicate that other parties often have been affected by the respondent’s drinking [3].

Earlier attempts to estimate harms to others than the drinker (for an overview see [4]) focussed on the social consequences, i.e. the “social victims of others’ drinking” [5], by asking questions about the extent of harm resulting from other peoples’ drinking, such as whether “someone who was drinking acted obnoxious or very unpleasant to you” or “someone neglected you because of their drinking”, and more serious problems such as “physical assault, sexual assault, accidents, and vandalization of property”. Similar survey approaches have been used in recent years with different and extended questionnaires, for example, in Canada [6], the USA [7], Norway [8], and in an international comparative study in Denmark, Finland, Iceland, Norway, Scotland, and Sweden [9].

In a comprehensive attempt, Laslett and colleagues [10] estimated more serious harms to others by using not only self-reported survey data but also registered data on road crash fatalities and hospitalizations, child abuse, and injuries sustained from interpersonal violence. These harms were derived using the population-attributable fraction methodology [11, 12], i.e. by estimating the proportions of these incidents which would disappear without any alcohol use [13]; for methodological details, see [14]. Using a similar approach, Rehm and colleagues [15] estimated alcohol-attributable morbidity and mortality caused by harms to others for transport injuries, violence, and low birth weight in the Member States of the European Union [16]. Most recently, systematic reviews and meta-analyses were used to estimate the national, regional, and global prevalence of fetal alcohol syndrome (FAS) [17] and fetal alcohol spectrum disorders (FASD) [18] worldwide.

By estimating morbidity and/or mortality of health harms to the newborn resulting from the mother’s drinking, and traffic and interpersonal violence fatalities to others than the drinker, we concentrate on the most severe consequences of harm to others [13]. Alcohol exposure in pregnancy is potentially associated with a broad range of physical defects; cognitive, behavioural, emotional and adaptive functioning deficits; and congenital anomalies in the newborn. Defects caused by intrauterine alcohol exposure are subsumed under the term FASD [19, 20], including symptoms of full-blown FAS which is considered the most severe and visible form of FASD [21]. Typical characteristics of FAS are facial anomalies and growth abnormalities. These features are clear-cut in childhood but less prominent during adolescence and adulthood. Damage to the brain due to alcohol exposure is mostly irreversible and associated with multiple co-morbidities and social consequences, such as dropping out of school, drug and alcohol misuse, abnormal sexual behaviour, and delinquency [22, 23].

Alcohol consumption is also a risk factor for both road traffic accidents and various kinds of aggression. Traffic accidents are among the leading causes of death and injury worldwide, causing an estimated 1.25 million deaths and more than 50 million injuries each year [24]. The risk of motor vehicle accidents is reported to increase progressively with increasing alcohol consumption [25]. Alcohol causes high levels of impairment in psychomotor performance [26] and impairs hazard perception [27], reaction time, attention, concentration, and hand-eye coordination [28, 29]. Acute intoxication also leads to a state of weakened attentional capacity and limited information processing ability, resulting in immediate reaction and confrontation, in some humans in the form of aggressive behaviour, rather than a reasoned response to everyday situations [30, 31].

Causality between alcohol use and road traffic collisions and fatalities, and between alcohol use and violence have long been established [32–34]. For both outcomes, there is an epidemiological and experimental research supporting the relationships, the latter with surrogate endpoints. Causality does not imply that all people drinking above a certain level will cause traffic collisions or become aggressive. It does mean, however, that alcohol is one factor in a combination of necessary antecedent conditions leading to the outcomes: in other words, in the absence of alcohol within these specific antecedent conditions, the outcome would not happen [35].

In sum, the present study aims to estimate some of the most severe harms that alcohol use may cause to people in Germany other than the drinker, namely FAS, FASD, road traffic fatalities caused by drunk drivers, and deaths resulting from alcohol-attributable interpersonal violence.

## Methods

### Fetal alcohol syndrome and fetal alcohol spectrum disorders

The estimations of FAS and FASD were based on the method proposed by Popova et al. [17] and Lange et al. [18]. This methodology estimates FAS and FASD indirectly using recent German prevalence data of alcohol use during pregnancy, as representative data on these diseases were not available for Germany. The incidences of FAS and FASD in countries with one or no empirical studies, including Germany, were thus predicted using data on the prevalence of alcohol use during pregnancy and estimations of the quotient for the average number of pregnant women who consumed alcohol per one case of FAS (1 in 13) or FASD (1 in 67) for countries with available data. The incidences of FAS or FASD were then predicted by applying this quotient to the country-specific prevalence of alcohol use during pregnancy. The Monte Carlo method was applied to derive the confidence intervals (CI) for the point estimates (for details on the methods, see [17, 18]).

### Data: prevalence of alcohol use during pregnancy

Data on the prevalence of alcohol use during pregnancy were obtained from the German Health Update Study (GEDA) [36] for the survey years 2009 (*n* = 21,262), 2010 (*n* = 22,050), and 2012 (*n* = 19,294). These data were pooled and weighted to represent the distribution of the general population on December 31, 2011. At the time of their interview, *n* = 374 women reported being pregnant, and of those women, 27.6% (95% CI 22.5%; 33.3%) reported having consumed alcohol during their pregnancy (calculations by C. Lange, data not published).

### Third-party road traffic fatalities

The estimations of the number of third-party road traffic and interpersonal violence fatalities were based on cause-of-death statistics and determined using the alcohol-attributable fraction methodology. The estimation of third-party traffic fatalities used the same methodology as the 2018 WHO Global Status Report on Alcohol and Health [37]. The AAF for motor vehicle accidents affecting people other than the driver (non-drivers (nd)) was estimated using Formula 1 based on the proportion of driver deaths attributable to alcohol consumption. The proportion of driver deaths attributable to alcohol was estimated based on driver deaths (*D*) as well as the AAFs for motor vehicle accidents affecting the drivers (*d*) by sex (indexed by *p*) and age (indexed by *i*). This method assumes that alcohol-attributable accidents affecting third parties and involving an intoxicated driver can be estimated based on the proportion of alcohol-attributable deaths among drivers. This method does not account for non-intoxicated drivers killed or injured by intoxicated drivers. The relative risks underlying traffic injuries were obtained from Corrao et al. [38].

1 $$ {\mathrm{AAF}}_{\mathrm{nd}}=\frac{\sum \limits_{p=1}^{pn}\sum \limits_{i=1}^{in}{D}_{p,i}\bullet {\mathrm{AAF}}_{p,i}}{\sum \limits_{p=1}^{pn}\sum \limits_{i=1}^{in}{D}_{p,i}} $$

### Interpersonal violence fatalities

The estimation of interpersonal violence fatalities due to alcohol was based on the method proposed by Cherpitel et al. [39]. Cherpitel and colleagues analysed the data from emergency department studies in 14 countries on the prevalence of patients’ self-reports of drinking within 6 h prior to a violence-related injury event, patients’ beliefs that the events would not have happened if they had not been drinking at the time, and patients’ perceptions that the perpetrators had been drinking. The AAFs were calculated based on the patients’ perceptions that their own drinking was causally related to the event and on their perceptions that the perpetrators had been drinking. The estimated AAF for others’ drinking was conservatively estimated at 14.9% only taking into account situations, where the respondents were sure of the causal attribution to drinking of others [39]. Standard errors and 95% CIs were calculated on the basis of the sample sizes and prevalence rates of the 14 countries resulting in a 95% CI from 12.5 to 17.4%. The underlying variability as measured in interquartile range between countries in the attributions in relevant items was 7.7% for the situation where the others were drinking but not oneself (median, 12.2%; 25 percentile, 8.0%; 75 percentile, 15.7%) and 29.5% for the situation with both self and others drinking (median, 24.9%; 25 percentile, 15.4%; 75 percentile, 44.9%).

### Data: road traffic and interpersonal violence fatalities

Data for 2014 on deaths due to road traffic and interpersonal violence fatalities were obtained from the cause-of-death statistics of the Federal Statistical Offices [40]. These statistics are based on diagnoses. Only those ICD version 10 codes which specifically address victims or third parties were selected. Codes for neglect and maltreatment (e.g. children or persons in need for care) were included as we consider it an interpersonal violence if a person dies due to these circumstances. The role of alcohol in neglect and maltreatment may differ from that in interpersonal violence, but due to the lack of evidence we assumed the same AAF as in other forms of assault. Table 1 summarizes the ICD-10 codes that were used in the present analysis.

**Table 1:** International Classification of Disease revision 10 codes used to estimate road traffic fatalities (victims) and assaults

Injury category	International Classification of Disease revision 10 codes
Road traffic fatalities^a^ (victims)	V021–V029, V031–V039, V041–V049, V092, V093, V123–V129, V133–V139, V143–V149, V194–V196, V203–V209, V213–V219, V223–V229, V233–V239, V243–V249, V253–V259, V263–V269, V273– V279, V283–V289, V294–V299, V304–V309, V314–V319, V324–V329, V334–V339, V344–V349, V354–V359, V364–V369, V374–V379, V384–V389, V394–V399, V404–V409, V414–V419, V424–V429, V434–V439, V444–V449, V454–V459, V464– V469, V474–V479, V484–V489, V494–V499, V504–V509, V514–V519, V524–V529, V534–V539, V544–V549, V554–V559, V564–V569, V574–V579, V584–V589, V594–V599, V604–V609, V614–V619, V624–V629, V634–V639, V644–V649, V654– V659, V664–V669, V674–V679, V684–V689, V694–V699, V704–V709, V714–V719, V724–V729, V734–V739, V744–V749, V754–V759, V764–V769, V774–V779, V784–V789, V794–V799, V803–V805, V811, V821, V830–V833, V840–V843, V850– V853, V860–V863, V870–V878, V892
Assaults	X85-Y09

^a^Including fatalities involving pedestrians, vehicle collisions, and other transport fatalities

## Results

### Fetal alcohol syndrome and fetal alcohol spectrum disorders

Based on the prevalence of alcohol use during pregnancy of 27.6%, the incidence of FAS in Germany was estimated at 0.41% (95% CI 0.24%; 0.63%) or 41 children with FAS per 10,000 live births. FASD was estimated at 1.77% (95% CI 1.35%; 3.26%) or 177 children with FASD per 10,000 live births per year. For the year 2014, when there were 714,927 live births in Germany, these estimates of FAS and FASD translate into 2930 (95% CI 1720; 4500) and 12,650 (95% CI 9650; 23,310) children born with FAS and FASD, respectively.

### Alcohol-attributable third-party road traffic fatalities

Table 2 provides the absolute numbers of third-party road traffic fatalities and the number of these deaths attributable to alcohol, by age group and gender. Overall, 1214 (95% CI 1141; 1287) of 2675 third-party road traffic accident deaths were estimated to be attributable to alcohol, representing 45.1% of the total mortality of third-party individuals due to road traffic fatalities in 2014.

**Table 2:** Third-party road traffic fatalities attributable to alcohol by age and gender in Germany in 2014

Sex and outcome	Age (years)				
	≤ 14	15–34	35–64	≥ 65	All ages
Men					
Deaths	37	704	833	476	2050
Alcohol-attributable deaths [95% CI]	17 [16, 18]	317 [298, 336]	375 [353, 398]	214 [202, 227]	924 [868, 979]
Women					
Deaths	18	147	187	292	644
Alcohol-attributable deaths [95% CI]	8 [8, 9]	66 [62, 70]	84 [79, 89]	132 [124, 139]	290 [273, 308]
Total					
Deaths	55	851	1020	768	2694
Alcohol-attributable deaths [95% CI]	25 [23, 26]	383 [360, 406]	460 [432, 487]	346 [325, 367]	1214 [1141, 1287]

*CI* confidence interval

### Alcohol-attributable interpersonal violence fatalities

Absolute numbers of interpersonal violence fatalities and the estimated number of these deaths which were alcohol-attributable are depicted by age group and gender in Table 3. In 2014, 368 people died from injuries sustained as a result of interpersonal violence, and 55 (95% CI 46; 64) of these deaths were estimated to be alcohol-attributable (26 for men, 29 for women).

**Table 3:** Interpersonal violence deaths attributable to alcohol by age and gender in Germany in 2014

Sex and outcome	Age (years)				
	≤ 14	15–34	35–64	≥ 65	All ages
Men					
Deaths	21	34	94	25	174
Alcohol-attributable deaths [95% CI]	3 [3, 4]	5 [4, 6]	14 [12, 16]	4 [3, 4]	26 [22, 30]
Women					
Deaths	13	41	89	51	194
Alcohol-attributable deaths [95% CI]	2 [2, 2]	6 [5, 7]	13 [11, 15]	8 [6, 9]	29 [24, 34]
Total					
Deaths	34	75	183	76	368
Alcohol-attributable deaths [95% CI]	5 [4, 6]	11 [9, 13]	27 [23, 32]	12 [10, 13]	55 [46, 64]

*CI* confidence interval

## Discussion

FAS and FASD in Germany were estimated at 0.41% (95% CI 0.24%; 0.63%) or 41 children and 1.77% (95% CI 1.35%; 3.26%) or 177 children per 10,000 live births per year, or 2900 (95% CI 1720; 4500) and 12,600 (95% CI 9650; 23,310) children, respectively. Based on the data for 2014, the numbers of alcohol-attributable third-party road traffic and interpersonal violence fatalities were estimated at 1214 (95% CI 1141 to 1287) and 55 (95% CI 46 to 64), respectively.

### Fetal alcohol syndrome and fetal alcohol spectrum disorders

Three points deserve to be mentioned concerning our estimations of alcohol-attributable FAS and FASD. First, FAS and FASD are not the only potential consequences of alcohol use during pregnancy. Other potential consequences include stillbirth [41], miscarriage [42], preterm birth [43], intrauterine growth restriction [44], and low birth weight [45]. Thus, FAS and FASD are only a portion of the damage which may be caused by alcohol use during pregnancy, albeit an important part.

Second, there are major methodological challenges in assessing the prevalence of alcohol use during pregnancy and the incidence of FAS and FASD [17, 18, 46]: (1) alcohol use during pregnancy is obtained through self-reports and is subject to reporting and recall bias, (2) validation—as is often done—of the diagnostic criteria for FAS/FASD in children already diagnosed with FAS/FASD uses no independent reference and leads to incorporation bias, and (3) the studies published so far do not rely on the same diagnostic guidelines or case definitions.

Third, while we used the best available methodology to estimate the prevalence of FAS/FASD in lack of a large representative study in Germany, it has to be acknowledged that using alcohol use as a predictor may lead to measurement bias, as the true underlying behaviour is likely binge drinking.

Only few studies exist which estimate the prevalence of FAS or FASD directly. For instance, studies in Italy estimated the prevalence of FAS at 37 to 74 per 10,000 children and for FASD at 203 to 405 per 10,000 children in 2004 [47]. For the years 2005–2007, estimates yielded a range of 65 to 101 per 10,000 children for FAS and 334 to 626 per 10,000 children for FASD [48]. A recent study on FASD in the USA reported higher rates, with conservative prevalence estimates ranging from 113 (95% CI 78 to 158) to 500 (95% CI 399 to 617) per 10,000 children and weighted estimates ranging from 311 (95% CI 161 to 540) to 985 (95% CI 575 to 1395) per 10,000 children [49]. The lack of original studies in Germany led Spohr and Steinhausen [50] to estimate the incidences of FAS and FASD based on international comparisons and analogies from studies conducted between 1970 and 2000 (FAS, 5 to 20 children per 10,000 live births [51, 52]; FASD, 40 to 60 children per 10,000 live births [51]). Hence, based on rather outdated estimates, the annual number of children in Germany with FAS was estimated at 600 to 1200 newborns and for the whole spectrum of FASD at 3000 to 4000 newborns per year. Based on the most recent data on the prevalence of alcohol use during pregnancy and the meta-analyses on the incidences of FAS and FASD conducted by Popova et al. [17] and Lange et al. [18], the present estimates for the incidences of FAS and FASD for Germany are substantially higher than earlier estimates (see also the discussion in *Lancet Public Health* [53–55]). A large-scale empirical study with active case ascertainment for Germany is necessary to determine incidences and prevalences and to determine which estimates are valid.

### Third-party road traffic fatalities

The present estimate of 1214 third-party road traffic fatalities, representing 45.1% of all such fatalities, is approximately 8 times higher than the estimate obtained when road traffic accident statistics, a breath alcohol level cut-off of 0.25 mg/L, and an alcohol per litre of blood (BAC) cut-off of 0.5 g/L for the perpetrator are used [56]. Road traffic accident statistics are very likely subject to underreporting due to deficits in the reporting of all cases, but particularly due to incorrect or missing information on the alcohol concentration of the person causing the accident. Since May 1, 1998, driving with a breath alcohol level above 0.25 mg/L or a BAC of above 0.5 g/L is classified as an administrative offence under § 24a of the Road Transport Law. However, drivers involved in road traffic fatalities who have an alcohol concentration below the above-stated limits but with a breath alcohol level of at least 0.15 mg/L or a BAC of at least 0.3 g/L are considered to have committed an alcohol-related offence [40]. Despite the legal basis for reporting alcohol involvement in road traffic accidents, the Federal Statistical Office has acknowledged that there is substantial underreporting of alcohol involvement in road traffic accident statistics as alcohol involvement is not routinely tested in all road traffic accidents.

The attributable fraction estimations for road traffic fatalities used in the present approach are limited by several factors related to the relative risks (RRs) which were used for these estimations. Firstly, the RRs were obtained from a meta-analysis where the underlying studies involved different countries, states, and cities [38] and where drinking contexts and the probability of injury after consuming alcohol may differ [57]. Secondly, the underlying RRs are based on the average daily alcohol consumption and thus do not take into consideration the frequency of binge drinking or the amount of alcohol consumed per binge drinking occasion which also may affect the RR estimations [58]. Finally, the number of third parties involved in road traffic fatalities may be underreported, potentially resulting in an underestimation of the number of alcohol-attributable third-party road traffic fatalities. Despite these limitations, the alcohol-attributable fraction methodology based on ICD codes reported in the cause-of-deaths statistics is considerably more reliable than the road traffic accident statistics and an appropriate cut-off.

### Interpersonal violence fatalities

Similarly to road traffic accident statistics, alternative statistics on alcohol-attributable interpersonal violence injuries and fatalities are available in Germany. The existing crime statistics, however, are subject to incomplete documentation of violent acts and are missing information on the degree of intoxication of the individuals. In 2014, the German Crime Statistics (Kriminalitätsstatistik) reported 250,000 suspects of violent acts where alcohol was involved. In 40,000 of 135,000 resolved violent acts, alcohol involvement could be verified [59]. In addition, BAC cut-offs for calculating alcohol-attributable proportions of violence to be used similarly to road traffic injuries and fatalities (see above) are not available. The potential causal pathways for alcohol leading to violence are multiple, and the likelihood of violence at a given level of drinking varies not only between cultures but is strongly influenced by contextual, situational, and personal factors [60].

Most importantly, while violence against women is particularly widespread [61], these incidents are highly underreported as they predominantly happen in intimate relationships [62, 63]. In 2014, the European Union Agency for Fundamental Rights published data on the prevalence and health effects of violence against women in Europe. According to this survey, one in ten women experienced some form of sexual violence since she was 15 years old (Germany 12%), some 22% experienced physical or sexual violence by a partner (Germany 22%), and psychological violence was reported by 43% of the interviewed women (Germany 50% [64].

In response to the widespread domestic and sexual violence against women, the WHO recently published evidence-based guidelines for responding to the issue in healthcare, training of healthcare providers, and health policy [65]. These recommendations, if successfully put into practice, may not only support those women most in need of help but may also improve the identification and monitoring of violence against women.

## Conclusions

The current study demonstrated the feasibility of estimating health harms to others using standard methodology from comparative risk assessments; however, while the study demonstrated general feasibility, there is still need for improvement. As indicated, active case ascertainment studies on the incidence and prevalence of each of FAS and FASD that overcome the methodological problems outlined above need to be conducted. Second, the existing guidelines for diagnosing FAS need to be put into practice by offering continuous training to paediatric and healthcare personnel. Road traffic accident statistics which include the involvement of alcohol have been available for Germany since 1995. These statistics may be used for the estimation of harms to others inflicted by intoxicated drivers if a suitable BAC cut-off is used for attributing the accident to alcohol. However, substantially lower estimates of alcohol-attributable road traffic fatalities compared to the AAF approach suggest that measures are needed to improve the recording of alcohol’s involvement and to reduce the underreporting of incidents. Similarly, the use of official crime statistics reporting alcohol’s involvement in interpersonal violence-related deaths is not recommended as there is presently no evidence for suitable cut-offs at which one may assume that an aggressive incident could have been avoided had alcohol not been involved.

The present findings of harms to others related to alcohol use during pregnancy, drunk driving, and interpersonal violence using the best available data reveal that alcohol may not only cause harm to the drinker but may also harm a substantial number of third parties. While the estimates across the three harm domains are not directly comparable, the results suggest that considering the short life expectancy among children with FAS/FASD alcohol use in pregnancy followed by road traffic fatalities account for significantly more harm to others than alcohol-related violence. The harmful effects of alcohol on others need to be recognized as a public health problem in the same way as are the harmful effects on the drinker or the costs to society [4]. Strategies to prevent harmful alcohol consumption are based on the growing literature on cost-effective measures to prevent alcohol-attributable harms [66–68]. Reductions in the overall rate of drinking are assumed to reduce heavy drinking and consequently alcohol-related harms. Although the evidence for this assumption is strong, measures such as pricing policies or limiting the marketing of alcoholic beverages are unpopular. However, targeted measures addressing particular populations at risk, such as women of childbearing age or road users, may help to reduce harms to others as well as harms to the drinker. For instance, screening and brief interventions in primary healthcare settings have been proven effective [69, 70] as well as cost-effective in reducing hazardous and harmful alcohol use [71]. Screening for alcohol use in all women of childbearing age in combination with preconception health promotion should become a routine in all primary healthcare settings.

Systematic reviews and meta-analyses have also shown that enforced legislative measures, including roadside breath testing and checkpoints as well as individually directed interventions to at-risk drinkers, are effective in reducing drink driving [67]. Finally, targeted interventions, such as training of bar service personnel [72] or teaching psychological harm reduction strategies [73], may help to reduce alcohol-related aggression. Although the mechanisms of alcohol causing aggression are not yet understood, there is evidence that alcohol facilitates aggression in individuals with the psychological predisposition by reducing the inhibiting forces and that alcohol indirectly contributes to aggression by causing cognitive, emotional, and psychological changes which reduce self-awareness and lead to a biased risk assessment [74] (for an overview, see [32]). Thus, interventions need to target persons with aggressive dispositions and teach them social interaction and interpretation skills so that they can remain non-violent when drinking.
